# The effect of vascular risk factor burden on the severity of COVID-19 illness, a retrospective cohort study

**DOI:** 10.1186/s12931-020-01510-0

**Published:** 2020-09-21

**Authors:** Houwei Du, Xiaobin Pan, Nan Liu, Junnian Chen, Xiaoling Chen, David J. Werring, Gareth Ambler, Xiaoqing Li, Ronghua Chen, Yixian Zhang, Huayao Huang, Feifei Lin, Pincang Xia, Chao Chen, Zhenyang Zheng, Sangru Wu, Hanhan Lei, Lei Gao, Mingxu Huang, Kexu Lin, Xiaoping Xu, Yukun Luo, Ziwen Zhao, Chen Li, Hailong Lin, Yu Lin, Zhenghui Huang, Rongxiang Cao, Limin Chen, On behalf of the Fujian Medical Team Support Wuhan for COVID-19

**Affiliations:** 1 Stroke Research Center, Department of Neurology, Fujian Medical University Union Hospital, 29 Xinquan Road, Gulou District, Fuzhou, 350001 China; 2 Institute of Clinical Neurology, Fujian Medical University, Fuzhou, China; 3 Department of Critical Care Medicine, Fujian Provincial Hospital South Branch, Fuzhou, China; 4 Department of Rehabilitation, Fujian Medical University Union Hospital, Fuzhou, China; 5 Department of Critical Care Medicine, Fujian Medical University Union Hospital, Fuzhou, China; 6 Department of Infectious Disease, Fujian Medical University Union Hospital, Fuzhou, China; 7 UCL Queen Square Institute of Neurology, London, UK; 8 Statistical Science, University College London, London, UK; 9 Fujian Center for Disease Control and Prevention, Fuzhou, China; 10 Department of Neurology, Fuzhou Second Hospital Affiliated to Xiamen University, Fuzhou, China; 11 Department of Thoracic Surgery, Fujian Medical University Union Hospital, Fuzhou, China; 12 Department of Emergency Medicine, Fujian Medical University Union Hospital, Fuzhou, China; 13 Department of Anesthesiology, Fujian Medical University Union Hospital, Fuzhou, China; 14 Department of Cardiology, Fujian Medical University Union Hospital, Fuzhou, China; 15 Department of Otolaryngology, Fujian Medical University Union Hospital, Fuzhou, China; 16 Department of Radiology, Fujian Medical University Union Hospital, Fuzhou, China; 17 Department of Colorectal Surgery, Fujian Medical University Union Hospital, Fuzhou, China; 18 Department of Respiratory Medicine, Fujian Medical University Union Hospital, Fuzhou, China

**Keywords:** Vascular risk factor, Coronavirus disease 2019, Prognosis

## Abstract

**Background:**

Patients with cardiovascular comorbidities are at high risk of poor outcome from COVID-19. However, how the burden (number) of vascular risk factors influences the risk of severe COVID-19 disease remains unresolved. Our aim was to investigate the association of severe COVID-19 illness with vascular risk factor burden.

**Methods:**

We included 164 (61.8 ± 13.6 years) patients with COVID-19 in this retrospective study. We compared the difference in clinical characteristics, laboratory findings and chest computed tomography (CT) findings between patients with severe and non-severe COVID-19 illness. We evaluated the association between the number of vascular risk factors and the development of severe COVID-19 disease, using a Cox regression model.

**Results:**

Sixteen (9.8%) patients had no vascular risk factors; 38 (23.2%) had 1; 58 (35.4%) had 2; 34 (20.7%) had 3; and 18 (10.9%) had ≥4 risk factors. Twenty-nine patients (17.7%) experienced severe COVID-19 disease with a median (14 [7–27] days) duration between onset to developing severe COVID-19 disease, an event rate of 4.47 per 1000-patient days (95%CI 3.10–6.43). Kaplan-Meier curves showed a gradual increase in the risk of severe COVID-19 illness (log-rank *P* < 0.001) stratified by the number of vascular risk factors. After adjustment for age, sex, and comorbidities as potential confounders, vascular risk factor burden remained associated with an increasing risk of severe COVID-19 illness.

**Conclusions:**

Patients with increasing vascular risk factor burden have an increasing risk of severe COVID-19 disease, and this population might benefit from specific COVID-19 prevention (e.g., self-isolation) and early hospital treatment measures.

## Background

The coronavirus disease 2019 (COVID-19) is a highly infectious viral respiratory illness caused by severe acute respiratory syndrome coronavirus 2 (SARS-CoV-2), which was first reported in December 2019 in Wuhan, China [1]. COVID-19 rapidly became a worldwide pandemic, affecting more than 28 million people [2]. Cardiovascular risk factors, including ischemic heart disease, hypertension and diabetes, are common in patients with COVID-19 infection, and associated with mortality [3, 4]. Cardiovascular diseases are a major public health challenge worldwide. For example, a population-based study with a large sample size showed that 6.63% (3070/46,285) of people had at least one major cardiovascular disease [5]. A global cohort study showed that 57,303 (39.4%) people had hypertension, 15,900 (10.2%) had diabetes and 26,691 (18.5%) reported low physical activity, and that mean body-mass index (BMI) was 25.7 (SD 5.3) kg/m^2^ [6]. Given the vast number of people living with prevalent vascular risk factors and the rapid global spread of the COVID-19 pandemic, it is essential to know how vascular risk factors influence the risk of developing severe COVID-19 disease. Most previous studies showed an association between vascular risk factors (such as hypertension and diabetes) and COVID-19 severity [7]. However, little is known about how patients stratified by burden of vascular risk factors differ from each other, and whether the number of vascular risk factors is associated with COVID-19 disease severity. We hypothesized that in patients infected with SARS-CoV-2 increasing vascular risk factor burden increases the risk of developing severe illness. We therefore investigated the association between the number of vascular risk factors and severe COVID-19 disease in this observational retrospective study.

## Methods

### Study design and participants

This is a single-center, retrospective, observational study done at Tumor Center of Union Hospital, Tongji Medical College, Huazhong University of Science and Technology (Wuhan, China), which is a designated hospital to treat patients with COVID-19 disease by national medical teams to support Wuhan. We analyzed admitted patients between 15 February and 14 March 2020, who had been diagnosed with COVID-19 disease based on World Health Organization interim guidance [8]. Laboratory confirmation of COVID-19 infection was performed by the local health authority as previously described [9].

### Data collection and outcome measures

We retrospectively reviewed the electronic medical records of 164 consecutive eligible patients with COVID-19 using a digital database. Two physicians (J.C and X.C) extracted the epidemiological, demographic, clinical, laboratory data on admission, and treatment data using a standardized data collection form. Data on the most intense level of oxygen treatment during hospitalization (nasal cannula, medical mask, high solution and invasive mechanical ventilation) was also collected. Two physicians (K.L and X.P) collected the outcome data, which was adjudicated by two senior physicians (H.D. and Y.L.) blinded to baseline demographics. Two physicians (G.L and R.C) reviewed the chest CT images blindly. In cases of disagreement, a consensus was reached after discussion with a senior respiratory physician (L.C.) and a radiologist (H.L). If data were missing or uncertain from the medical records, we obtained and clarified data by direct communication with attending doctors and other healthcare providers.

Definitions of vascular risk factors were based on previous literature [6, 10–13] as follows. Hypertension was defined as documented systolic blood pressure ≥ 140 mmHg and/or diastolic blood pressure ≥ 90 mmHg, or by patients’ self-reported diagnosis of hypertension and/or by the treatment of antihypertensives [10, 11]. Diabetes was defined as fasting blood glucose ≥7.0 mmol/L and/or a previous diagnosis of diabetes and/or by the treatment of antidiabetic drugs [10]. Dyslipidaemia was defined as TC ≥6.22 mmol/L and/or TG ≥2.26 mmol/L and/or HDL-C < 1.04 mmol/L and/or LDL-C ≥ 4.14 mmol/L [10]. We classified patients as having atrial fibrillation (AF) if they had been diagnosed with AF by electrocardiogram at admission or during hospital stay [11]. Current smoking (≥1 cigarette per day) and regular drinker (any dose of alcohol, ≥1 time per week) were defined by patients’ self-report [12]. Physical inactivity was defined as less than 150 min per week of moderate activity or less than 75 min per week of vigorous activity [13], and overweight as body mass index (BMI) ≥25 kg/m^2^ [10]. We counted and aggregated the following vascular risk factors to construct a vascular risk factor score in each patient: hypertension, diabetes, dyslipidemia, atrial fibrillation, current smoker, overweight and physical inactivity. These factors are considered well-documented risk factors for cardiovascular diseases, including cerebrovascular disease [6, 10–14]. Our primary outcome was severe COVID-19 disease defined as fever or suspected respiratory infection, plus one of: respiratory rate > 30 breaths/min; severe respiratory distress; or SPO_2_ ≤ 93% on room air based on the interim guidance of the World Health Organization [8]. Our secondary outcome was death, which was limited by the duration of our observation period. All the authors agreed on the study protocol and reviewed the manuscript.

### Statistical analysis

Continuous data were summarized using means with standard deviations or medians with interquartile ranges (IQR), and categorical data were summarized as counts with percentages. We used the t-test or Mann-Whitney test to compare differences in continuous variables, and the chi-square test or Fisher’s exact test to compare differences in categorical variables. We calculated the absolute event rate per 1000 patient-days for severe COVID-19 illness and death during our observation period. We also compared event rates during our observation period stratified by the number of vascular risk factors (none, 1, 2, 3, or ≥ 4 risk factors). Kaplan-Meier curves depicted the risk for outcome events stratified by the number of vascular risk factors (none, 1, 2, 3, or ≥ 4 risk factors). We used univariable Cox regression analysis to evaluate the association between vascular risk factor burden and severe COVID-19 pneumonia. For multivariable analysis, we chose variables based on previous findings and clinical constraints to avoid overfitting the Cox regression model [15]. We therefore included chronic obstructive pulmonary disease (COPD), cardio-cerebrovascular disease, tumor, renal impairment, decreased leucocytes, decreased lymphocytes, increased lactate dehydrogenase (LDH) and chest CT findings separately as potential confounders, in addition to age and sex in the regression model. We also performed sensitivity analyses using the E-value approach, where the E-value is defined as the minimum strength of association on the risk ratio scale that an unmeasured confounder would need to have with both the exposure and the outcome, conditional on the measured covariates, to fully explain away a specific exposure–outcome association [16]. We calculated the E-value using an online E-value calculator (https://mmathur.shinyapps.io/evalue/). All analyses were performed using STATA 12.0 (StataCorp LP, College Station, TX) and SPSS for Windows (SPSS 25.0, IBM, Inc., Chicago, IL, USA).

## Results

In this analysis, we included 164 consecutive patients (61.8 ± 13.6 years) who had COVID-19 disease between 15 February and 14 March 2020. Sixteen (9.8%) patients had no vascular risk factor, whereas 38 (23.2%) had 1, 58 (35.4%) had 2, 34 (20.7%) had 3, and 18 (10.9%) had ≥4 risk factors. The demographics, clinical and radiological characteristics in patients with severe COVID-19 illness and non-severe COVID-19 illness are shown in Table 1. Patients with severe COVID-19 disease were similar to those with non-severe illness regarding their previous history of diabetes, COPD, tumor, treatment with immunosuppressives, exposure to wet seafood market, and onset symptoms. Patients with severe disease were older (71.0 ± 12.8 vs 59.8 ± 12.9, *p* < 0.001), more likely to be male (20 [69.0%] vs 64 [47.4%], *p* = 0.04), and more likely to have pre-existent cardio-cerebrovascular disease (8 [27.6%] vs 15 [11.1%], *p* = 0.02). Regarding vascular risk factors, patients with severe COVID-19 disease were more likely to have hypertension (13 [44.8%] vs 39 [28.9%], *p* = 0.09), dyslipidemia (18 [62.1%] vs 45 [33.3%], *p* = 0.004), overweight (14 [48.3%] vs 41 [30.4%], *p* = 0.064) and physical inactivity (26 [89.7%] vs 77 [57.0%], *p* = 0.001). The severe COVID-19 event rate increased with the number of vascular risk factors (Table 2). Looking at routine blood test findings, patients with severe COVID-19 disease were more likely to have decreased leucocytes (17.2% vs 4.4%, *p* = 0.01), decreased lymphocytes (65.5% vs 26.7%, *p* < 0.001), and increased lactic dehydrogenase (LDH) (58.6% vs 24.6%, p < 0.001). Regarding chest computed tomography (CT) findings, patients with severe COVID-19 illness were more likely to be bilaterally affected.

**Table 1:** Demographic, clinical, laboratory, radiological characteristics, treatment and outcome between severe and non-severe COVID-19 pneumonia

	Total (*n* = 164)	Severe (*n* = 29)	Non-severe (*n* = 135)	*P*
Age, (y) mean ± SD	61.8 ± 13.6	71.0 ± 12.8	59.8 ± 12.9	< 0.001
Male, n (%)	84 (51.2)	20 (69.0)	64 (47.4)	0.04
Current smoker, n (%)	17 (10.4)	2 (6.9)	15 (11.1)	0.73
Regular drinker, n (%)	3 (1.8)	0	3 (2.2)	> 0.99
Hypertension, n (%)	52 (31.7)	13 (44.8)	39 (28.9)	0.09
Diabetes, n (%)	31 (18.9)	7 (24.1)	24 (17.8)	0.43
Dyslipidemia, n (%)	63 (38.4)	18 (62.1)	45 (33.3)	0.004
Atrial fibrillation, n (%)	10 (6.1)	4 (13.8%)	6 (4.4%)	0.14
Overweight, n (%)	55 (33.5)	14 (48.3)	41 (30.4)	0.06
Physical inactivity, n (%)	103 (62.8)	26 (89.7)	77 (57.0)	0.001
COPD, n (%)	12 (7.3)	4 (13.8)	8 (5.9)	0.28
Cardio-cerebrovascular disease, n (%)	23 (14.0)	8 (27.6)	15 (11.1)	0.020
Renal impairment, n (%)	25 (15.2)	10 (34.5)	15 (11.1)	0.001
Digestive disease, n (%)	15 (9.1)	1 (3.4)	14 (10.4)	0.41
Immunosuppresives, n (%)	3 (1.8)	1 (3.4)	2 (1.5)	> 0.99
Tumor, n (%)	13 (7.9)	3 (10.3)	10 (7.4)	0.88
Wet market exposure, n (%)	2 (1.2)	1 (3.4)	1 (0.7)	0.79
Clinical symptoms				
Fever, n (%)	115 (70.1)	20 (69.0)	95 (70.4)	0.88
Dry cough, n (%)	104 (63.4)	19 (65.5)	85 (63.0)	0.80
Productive cough, n (%)	23 (14.0)	2 (6.9)	21 (15.6)	0.36
Fatigue, n (%)	57 (34.8)	9 (31.0)	48 (35.6)	0.64
Muscle or joint ache, n (%)	21 (12.8)	2 (6.9)	19 (14.1)	0.46
Thoracalgia, n (%)	31 (18.9)	6 (20.7)	25 (18.5)	0.79
Sore throat, n (%)	23 (14.0)	4 (13.8)	19 (14.1)	> 0.99
Diarrhea, n (%)	13 (7.9)	4 (13.8)	9 (6.7)	0.36
Catarrh, n (%)	6 (3.7)	0	6 (4.4)	0.59
Anorexia, n (%)	48 (29.3)	8 (27.6)	40 (29.6)	0.83
Short of breath, n (%)	65 (39.6)	15 (51.7)	50 (37.0)	0.14
Headache, n (%)	19 (11.6)	3 (10.3)	16 (11.9)	> 0.99
Total symptoms (IQR)	3 [2–4]	3 [2–4]	3 [2–4]	0.94
Routine blood examinations				
Decreased leucocytes, n (%)	11 (6.7)	5 (17.2)	6 (4.4)	0.01
Decreased lymphocytes, n (%)	55 (33.5)	19 (65.5)	36 (26.7)	< 0.001
Decreased hemoglobin, n (%)	42 (25.6)	15 (51.7)	27 (20.0)	< 0.001
Decreased platelets, n (%)	14 (8.5)	7 (24.1)	7 (5.2)	0.001
Increased ALT or AST, n (%)	58 (35.4)	14 (18.3)	44 (32.6)	0.11
Increased LDH, n (%)	50 (30.7)	17 (58.6)	33 (24.6)	< 0.001
Complications				
Acute stroke, n (%)	3 (1.8)	3 (10.3)	0	0.005
Shock, n (%)	3 (1.8)	3 (10.3)	0	0.005
CT findings, n (%)				0.04
Unilateral pneumonia, n (%)	26 (15.9)	3 (10.3)	23 (17.0)	
Bilateral pneumonia, n (%)	86 (52.4)	11 (37.9)	75 (55.6)	
Multiple mottling and ground-glass opacity, n (%)	52 (31.7)	15 (51.7)	37 (27.4)	
Treatment				
Oxygen therapy, n (%)				< 0.001
Nasal cannula, n (%)	79 (48.2)	16 (55.2)	63 (46.7)	
Medical mask, n (%)	5 (3.0)	5 (17.2)	0	
High solution, n (%)	5 (3.0)	5 (17.2)	0	
Invasive ventilation, n (%)	3 (1.8)	3 (10.3)	0	
Glucocorticoid, n (%)	20 (12.2)	9 (31.0)	11 (8.1)	0.001
Antibacterial, n (%)	117 (71.8)	23 (82.1)	94 (69.6)	0.18
Antivirus, n (%)	158 (96.3)	27 (93.1)	131 (97.0)	0.63
Chinese traditional medicine, n (%)	156 (95.1)	26 (89.7)	130 (96.3)	0.30
Outcomes Cured at discharge, n (%)	103 (62.8)	9 (31.0)	94 (69.6)	< 0.001
Death, n (%)	6 (3.7)	6 (20.7)	0	< 0.001

Tumor was defined by patients’ self-report of having a history of a malignant tumor. Decreased means below the lower limit of the normal range and increased means over the upper limit of the normal range

*Abbreviations*: *COVID-19* coronavirus disease 2019, *SD* Standard deviation, *COPD* Chronic obstructive pulmonary disease, *IQR* Interquartile range, *ALT* Alanine transaminase (U/L; normal range 0–40), *AST* Alanine aminotransferase (U/L; normal range 0–40), *LDH* Lactate dehydrogenase (U/L; normal range 109–245, data available in 163 patients), *CT* Computed tomography, Leucocytes (× 10^9^/L; normal range 3.5–9.5), Lymphocytes (× 10^9^/L; normal range 1.1–3.2), Platelets (× 10^9^/L; normal range 125.0–350.0), Hemoglobin (g/L; normal range 130.0–175.0)

**Table 2:** Number of cardiovascular risk factors and severe COVID-19 events

Vascular risk factor number	Severe COVID-19 event, n (%)*
No risk factor *n* = 16	1 (6.3)
One risk factor *n* = 38	2 (5.3)
Two risk factors *n* = 58	8 (13.8)
Three risk factors *n* = 34	8 (23.5)
Four risk factors *n* = 15	8 (53.3)
Five risk factors *n* = 3	2 (66.7)

Vascular risk factors include hypertension, diabetes, dyslipidemia, atrial fibrillation, current smoker, overweight and physical inactivity

*Abbreviations*: *COVID-19* coronavirus disease 2019

* *P* < 0.001 (chi-square test)

**Fig. 1:**
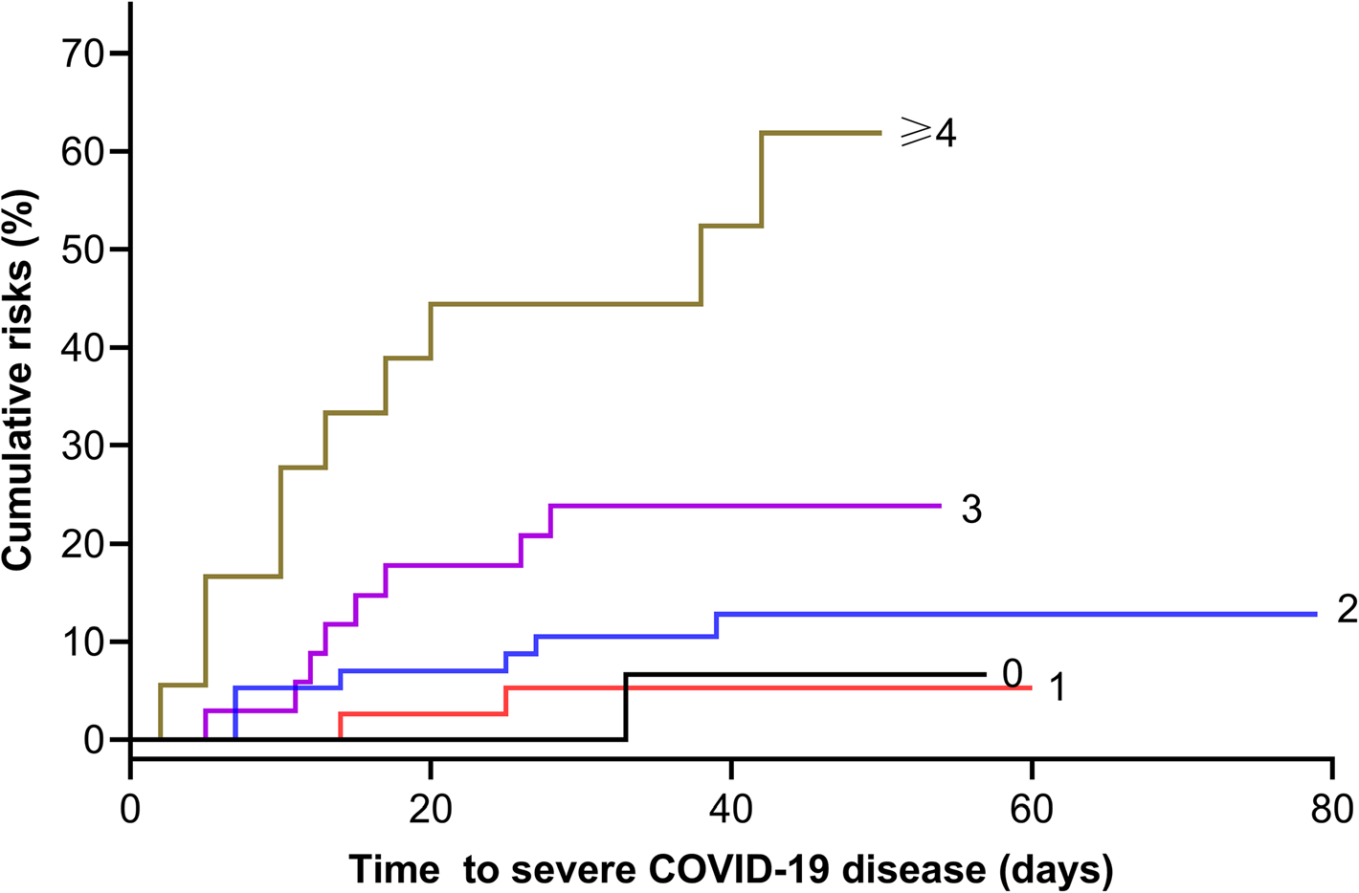
Cumulative probability of severe COVID-19 disease stratified by the number of vascular risk factors. Figure legend: COVID-19 = coronavirus disease 2019

The median follow-up time was 42 days [IQR 35–49], providing 6493 patient-days of data. One hundred and three (62.8%) patients were cured at discharge at the end of our observation period. Twenty-nine patients (17.7%) experienced severe COVID-19 illness with a median (14 [7–27] days) duration between symptom onset to developing a severe disease, an event rate of 4.47 per 1000-patient days (95%CI 3.10–6.43). Six patients (3.7%) died during hospitalization, an event rate of 0.84 per 1000-patient days (95%CI 0.38–1.88). Kaplan-Meier curves showed that the risk of severe COVID-19 illness tended to increase with the number of vascular risk factors (log rank *P* < 0.001, Fig. 1). In univariable analysis, the risk of severe COVID-19 increased with increasing vascular risk factor burden (unadjusted HR 2.06, 95%CI 1.51–2.80). After adjustment for age and sex, the vascular risk factor burden remained significantly associated with severe COVID-19. (adjusted OR 1.55 95%CI 1.09–2.21, Table 3**).** This relationship did not change after additional confounder adjustment for, separately, COPD, cardio-cerebrovascular disease, tumor and renal impairment, in addition to age and sex (Table S1). Separate adjustment for routine blood examinations and chest CT findings, in addition to age and sex, did not alter our results (Table S2). Sensitivity analyses revealed that it would need moderately robust unobserved confounding to render the exposure-outcome association null. (Table 3**,** Table S1 and Table S2). For example, an E-value of 2.12 for the estimate (Table 3**,** and Figure S1) indicates that the observed risk ratio of 2.12 could only be explained away by an unmeasured confounder that was associated with both the exposure and the outcome by risk ratios of more than two, above and beyond the measured confounders, but weaker confounding could not do so [16].

**Table 3:** Association between vascular risk factor burden and severe COVID-19 illness and sensitivity analyses using the E-value approach

	Unadjusted		Age- and sex- adjusted	
E-value	NA		2.12	
Number of vascular risk factor	HR (95% CI)	*p* value	HR (95% CI)	*p* value
	2.06 [1.51–2.80]	< 0.001	1.55 [1.09–2.21]	0.01

Abbreviations: *COVID-19* coronavirus disease 2019, *HR* hazard ratio

Vascular risk factors include hypertension, diabetes, dyslipidemia, atrial fibrillation, current smoker, overweight and physical inactivity

E-values represent the minimum strength of association an unmeasured confounder would have to possess between both the exposure and the outcome in order to reduce the observed association between the exposure and outcome to 1 (no association) on the relative scale

## Discussion

Our most important finding is that in COVID-19 patients increasing vascular risk factor burden is associated with an increasing risk of experiencing severe COVID-19 illness. The seven included vascular risk factors are generally readily available at hospital admission, which may help clinicians to identify patients with poor prognosis at an early stage.

Previous studies suggested patients with mild COVID-19 disease were at low risk for an unfavorable outcome than those with severe COVID-19 disease [4, 15]. Therefore, identifying risk factors for severe COVID-19 illness is essential to ensure timely management. In our cohort, symptoms at COVID-19 onset were not significantly different between patients with and without severe COVID-19 illness, suggesting that onset symptoms were not of prognostic relevance. The association between total baseline vascular risk factor burden and severe COVID-19 illness in our cohort may inform the development of future risk stratification models.

Our data showed that patients with severe COVID-19 illness were more likely to have a baseline ischemic cardiac or cerebrovascular disease (8 [27.6%] vs 15 [11.1%]). This result is line with findings of previous descriptive studies [3, 17]. Previous studies also showed individual vascular risk factors such as hypertension and diabetes were more frequent in severe COVID-19 patients [3, 14]. Moreover, data from Italy and the United States confirmed increased mortality rates in patients with comorbidities, particularly the older population with pre-existent cardiovascular conditions [18, 19]. Our findings showed vascular risk factors were prevalent among COVID-19 patients. Only sixteen (9.8%) patients had no vascular risk factor, whereas two-thirds of patients had more than one risk factor. Patients with increasing vascular risk factor burden in our cohort were older (*p* < 0.001), having a high risk for the severe COVID-19 disease, and consequently underwent more aggressive ventilation. To the best of our knowledge, the association between total vascular risk factor burden and the severity of COVID-19 has not been systematically investigated before. Our data therefore expands upon previous findings of individual comorbidities as risk factors for severe COVID-19 disease and may be relevant for important public health and clinical decisions regarding the management of the current COVID-19 and future pandemics.

Among the vascular risk factors associated with severe COVID-19, being overweight showed a strong association [20]. People with obesity around the world are at high risk for severe COVID-19 illness [21]. For example, a previous study has shown that overweight patients had a 1.84-fold higher risk of developing severe COVID-19 (95% CI 0.99–3.43) than normal-weight patients [22]. Moreover, our data indicate that patients with physical inactivity are more likely to have severe COVID-19 illness. The relationship between these lifestyle factors and COVID-19 severity warrants further studies for clarification, but in the meantime our findings further strengthen the idea that the population at risk of COVID-19 should keep exercising at home during the quarantine. Moreover, considering the strict isolation strategies to curb virus spreading, the effect of physical inactivity on the potential risk of the cardio-cerebrovascular events needs further urgent investigation worldwide.

Whether some antihypertensives could increase the susceptibility to developing severe forms of COVID-19 remains uncertain. SARS-CoV-2 is linked to the renin-angiotensin system (RAS), which plays an essential role in regulating blood pressure and the pathophysiology of cardiovascular disease [23]. Patients with pre-existing vascular risk factor burden such as hypertension and diabetes were likely to be treated with angiotensin-converting enzyme inhibitors (ACEI) or angiotensin receptor blockers (ARB). Both ACEI and ARB can upregulate ACE2, facilitating the SARS-CoV-2 entry into pneumocytes, and might cause exacerbation of the underlying disease [24]. However, available clinical evidence does not confirm an association between the use of ACEI or ARB and the poor outcomes of COVID-19 [7, 25, 26]. Long-term studies in larger numbers of hospitalized COVID-19 patients receiving ACEI and ARB therapy are needed to address this question.

The underlying mechanisms of the association between vascular risk factor burden and COVID-19 severity remain unclear. Immobilization and high body mass were found to be risk factors for pulmonary embolism [27, 28], which were prevalent in severe COVID-19 disease [29, 30]. Other possible explanations might include exaggerated systemic inflammation or a “cytokine storm”. For example, metabolic risk factors such as obesity may increase the inflammation of the lung parenchyma and compromise the pulmonary function [31, 32].

Our findings suggest that clinicians should pay close attention to the warning signs and symptoms of cardiac or cerebrovascular events in COVID-19 patients with high vascular risk factor burden. Establishing a multi-disciplinary medical team of cardiologists, neurologists, respiratory specialists, and infectious disease specialists to offer rapid evaluation and acute treatment in COVID-19 is urgently needed.

We acknowledge limitations. First, this is a retrospective study conducted at a single-centered hospital with limited sample size. Our findings need to be validated in further large sample-sized studies. Second, some vascular risk factors were self-reported, so their prevalence may be underestimated. However, previous studies showed that self-reports and hospital records were highly consistent for vascular disease [5, 33]. Lastly, lack of information on some other vascular risk factors (i.e., air pollution, depression, and sleep disorder) may cause inaccuracy in risk estimates based on the number of these risk factors.

## Conclusions

Vascular risk factor burden is associated with the course of COVID-19 infection; patients with an increasing number of vascular risk factors have an increasingly high risk of severe COVID-19 disease and might benefit from specific COVID-19 prevention (e.g., self-isolation) and early hospital treatment measures. A better understanding of the pathophysiological mechanisms causing severe COVID-19 disease is needed to develop new treatment strategies for improving outcomes and reducing mortality in this high-risk population.

## Supplementary information

**Additional file 1: Table S1.** Association between vascular risk factor burden and severe COVID-19 illness adjusted for comorbidity, and sensitivity analyses using the E-value approach. **Table S2.** Association between vascular risk factor burden and severe COVID-19 illness adjusted for laboratory and chest CT findings, and sensitivity analyses using the E-value approach. **Figure S1.** Bias Plots

## Data Availability

The datasets used and/or analyzed during the current study are available from the corresponding author on reasonable request (Email: houweidu@fjmu.edu.cn).

## Abbreviations

COVID: Coronavirus disease 2019
WHO: World Health Organization
SD: Standard deviation
COPD: Chronic obstructive pulmonary disease
IQR: Interquartile range
ALT: Alanine transaminase
AST: Alanine aminotransferase
LDH: Lactate dehydrogenase
CT: Computed tomography
HR: Hazard ratio
