# Supplementary material for: The effect of vascular risk factor burden on the severity of COVID-19 illness, a retrospective cohort study
Source: Respir Res. 2020 Sep 21;21:241. doi: 10.1186/s12931-020-01510-0 (PMC7503438; doi:10.1186/s12931-020-01510-0)

**SUPPLEMENTAL MATERIAL**

Supplemental Table S1

Supplemental Table S2

Supplemental Figure S1 Bias Plots

**Table S1: Association between vascular risk factor burden and severe COVID-19 illness adjusted for comorbidity, and sensitivity analyses using the E-value approach**

|  | Model 1 | | Model 2 | | Model 3 | | Model 4 | |
| --- | --- | --- | --- | --- | --- | --- | --- | --- |
| E-value | 2.21 | | 2.15 | | 2.10 | | 1.98 | |
| Number of  vascular risk factors | Adjusted HR | P | Adjusted HR | P | Adjusted HR | P | Adjusted HR | P |
|  | 1.61 [1.12-2.32] | 0.01 | 1.57 [1.09-2.25] | 0.015 | 1.54 [1.08-2.19] | 0.02 | 1.46 [1.01-2.10] | 0.04 |

Abbreviations: COVID-19 = coronavirus disease 2019; HR = hazard ratio;

Model 1: adjusted for age, sex and chronic obstructive pulmonary disease; Model 2: adjusted for age, sex and cardio-cerebrovascular diseae; Model 3: adjusted for age, sex and tumor; Model 4: adjusted for age, sex and renal impairment;

E‐values represents the minimum strength of association an unmeasured confounder would have to possess between both the exposure

and the outcome in order to reduce the observed association between the exposure and outcome to 1 (no association) on the relative scale.

For example, for an E‐Value of 2.21, an unmeasured confounder would have to have relative risk of association of ≥2.21 for the outcome *and* a relative risk of association of ≥ 2.21 to make the exposure‐outcome association null.

**Table S2: Association between vascular risk factor burden and severe COVID-19 illness adjusted for laboratory and chest CT findings, and sensitivity analyses using the E-value approach**

|  | Model 5 | | Model 6 | | Model 7 | | Model 8 | |
| --- | --- | --- | --- | --- | --- | --- | --- | --- |
| E-value | 2.07 | | 2.09 | | 2.25 | | 2.12 | |
| Number of  vascular risk factor | Adjusted HR | P | Adjusted HR | P | Adjusted HR | P | Adjusted HR | P |
|  | 1.52 [1.06-2.17] | 0.02 | 1.53 [1.07-2.19] | 0.02 | 1.64 [1.13-2.38] | 0.009 | 1.55 [1.09-2.21] | 0.02 |

Abbreviations: COVID -19 = coronavirus disease 2019; CT = Computed tomography;

Model 5: adjusted for age, sex and decreased leucocytes; Model 6: adjusted for age, sex and decreased lymphocytes; Model 7: adjusted for age, sex and increased lactic dehydrogenase; Model 8: adjusted for age, sex and chest CT findings

E‐values represents the minimum strength of association an unmeasured confounder would have to possess between both the exposure

and the outcome in order to reduce the observed association between the exposure and outcome to 1 (no association) on the relative scale.

For example, for an E‐Value of 2.07, an unmeasured confounder would have to have relative risk of association of ≥2.07 for the outcome *and* a relative risk of association of ≥ 2.07 to make the exposure‐outcome association null.

**Figure S1: Bias Plots**

Figure legend: Bias plots represent varying levels of unobserved confounder‐exposure association and unobserved confounder‐outcome association necessary to render observed exposure‐outcome association null for cardiovascular risk factor per point increase.


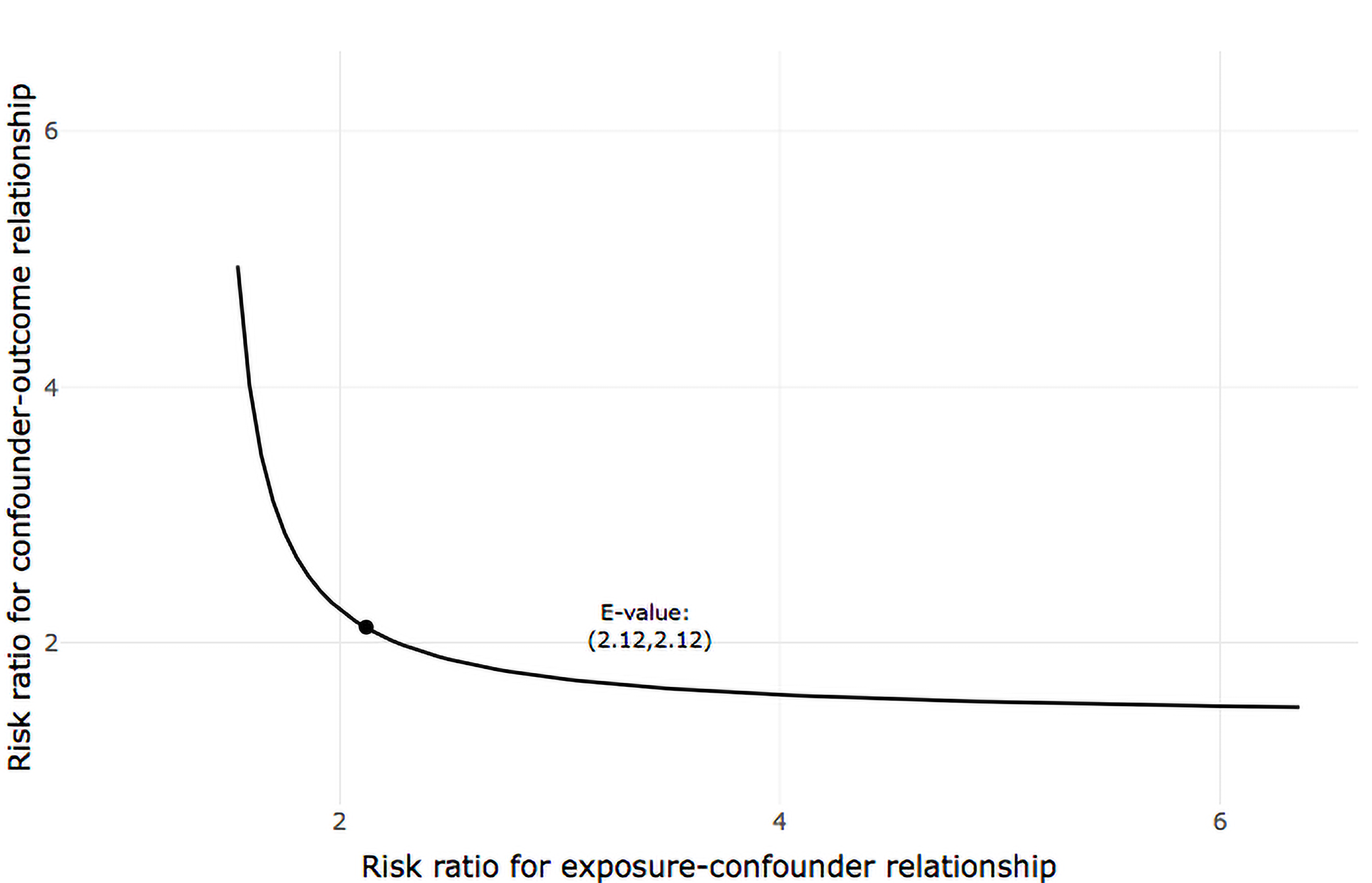

Supplement: Supplementary file 1 — Additional file 1: Table S1. Association between vascular risk factor burden and severe COVID-19 illness adjusted for comorbidity, and sensitivity analyses using the E-value approach. Table S2. Association between vascular risk factor burden and severe COVID-19 illness adjusted for laboratory and chest CT findings, and sensitivity analyses using the E-value approach. Figure S1. Bias Plots [file 12931_2020_1510_MOESM1_ESM.docx]
